# Unravelling the genetic diversity of RIPR: a key invasion protein in *Plasmodium vivax* malaria

**DOI:** 10.1017/S0031182026102157

**Published:** 2026-05

**Authors:** Jose Cebrian-Carmona, Lilia Gonzalez-Ceron, Veronica Valero-Galvez, Jose Antonio Garrido-Cardenas, Concepcion Mesa-Valle

**Affiliations:** 1 Departamento de Biología y Geología, Universidad de Almería, Almería, Spain; 2 Centro Regional de Investigación en Salud Pública, Instituto Nacional de Salud Pública, Tapachula, Mexico

**Keywords:** amino acids, neutrality tests, nucleotide diversity, *Plasmodium vivax*, *pvripr*, *Z* test

## Abstract

*Plasmodium vivax* RH5-interactive putative protein (RIPR) is present in micronemes and might participate in erythrocyte invasion. In this study, the polymorphism of *pvripr* gene was examined. In 7 *P. vivax* isolates from southern Mexico (SM), the *pvripr* gene was amplified and sequenced. Other *pvripr* sequences were retrieved from PlasmoDB; 15 from SM and 78 from other locations. The genetic parameters, deviation from neutrality, *Z* selection test, haplotype networks, *F*_ST_ index of differentiation and the amino acid substitutions were analysed. The phylogenetic tree of 22 SM isolates suggested 2 genetic groupings, and these isolates had lower nucleotide diversity (*n* = 22; π = 0.0007, Hd = 0.671) than Colombian, Peruvian and Asian parasites (π = 0.0011–0.0017, Hd > 0.9). The haplotype network of Mexican *pvripr* had haplotypes separated by 1–13 mutational steps among them, and a complex structure was observed in parasites from Peru, Colombia and China. No significant deviation from neutrality was estimated; however, Tajima’s D values across 3′ segment were negative in parasites from all locations. The *Z* test was significantly positive only for Latin American parasites, and the codon-level selection analysis showed several codons with *dN–dS* positive values in East Asia and Southeast Asia parasites. Given the lack of long-term *in vitro* culture for *P. vivax, in silico* epitope mapping across global sequences provides a pragmatic, hypothesis-generating framework. Globally, 12 polymorphic residues involved charged amino acid changes, many within predicted B-cell epitopes, consistent with immune-mediated selection on PvRIPR.

## Graphical abstract

**Figure:**
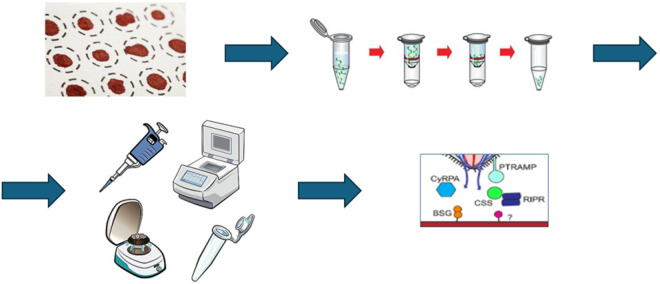

## Introduction

Globally, malaria remains a significant public health challenge, with 282 million cases reported in 2024 (World Health Organization, 2025). In the American continent, *Plasmodium vivax* exhibits high prevalence, and in recent years, there has been a notable surge in both the incidence and severity of this affliction (World Health Organization, 2025). To reduce malaria’s impact, vaccine development focusing on merozoite proteins is crucial. These vaccines aim to lower parasitemia and disease severity and, ultimately, might reduce transmission, supporting malaria elimination efforts. Additionally, merozoite proteins serve as valuable biomarkers for serological surveillance (Ellis et al., 2010; França et al., 2016).

Successful *Plasmodium* merozoite invasion into erythrocytes requires a complex interplay of parasite ligands and host cell receptors (Gilson and Crabb, 2009). While the invasion mechanisms of the highly virulent *Plasmodium falciparum* and the more prevalent *P. vivax* differ, both species rely on intricate protein interactions. In *P. falciparum*, a trimeric complex formed by reticulocyte-binding protein homolog 5 (PfRH5), RH5-interactive putative protein (PfRIPR) and Cysteine-rich protective antigen (PfCyRPA) is essential for erythrocyte invasion. This complex is a potent immunogen, making it a promising target for malaria vaccine development. While the precise homologous gene of PfRh5 in *P. vivax* and *Plasmodium knowlesi* remains elusive, homologues of PfRIPR and PfCyRPA have been identified (Chan et al., 2020). A recent investigation established the indispensability of PkRIPR and PkCyRPA for the viability of *P. knowlesi* (Knuepfer et al., 2019). Intriguingly, PkRIPR forms a tri-protein complex with thrombospondin-related apical merozoite protein and the small cysteine-rich secreted protein rather than binding to PkCyRPA (Knuepfer et al., 2019; Chan et al., 2020). The conditional disruption of any component within this tri-protein complex resulted in merozoites capable of adhering to human erythrocytes but unable to initiate invasion. These findings imply that RIPR and CyRPA play distinctive roles in *P. knowlesi* compared to *P. falciparum* (Knuepfer et al., 2019; Kepple et al., 2021).

PfRIPR plays an essential role in erythrocyte invasion and is considered a promising vaccine target, as antibodies against RIPR and other complex components can block invasion (Healer et al., 2019; Nagaoka et al., 2020; Patarroyo et al., 2020). In *P. falciparum*, RIPR forms a critical trimeric complex with RH5 and CyRPA that mediates invasion through interaction with the erythrocyte receptor basigin (BSG/CD147) (Knuepfer et al., 2019). In contrast, *P. knowlesi* utilizes a distinct RIPR-containing complex (TRAMP/CSS/RIPR), and knockout of *pkripr* results in non-viable parasites, indicating an essential role in parasite development (Knuepfer et al., 2019). Although polyclonal antibodies targeting basigin reduce *Plasmodium* invasion, the dependence on this pathway varies among species (Knuepfer et al., 2019). Notably, *P. vivax* RIPR does not appear to directly bind to basigin, and its precise function and binding partners remain unclear (Wong et al., 2019; Ndwiga et al., 2021). Given the close evolutionary relationship between *P. knowlesi* and *P. vivax*, and the genomic characteristics of *pvripr* as a single-exon gene encoding a microneme protein on chromosome 5, further studies are required to elucidate the role of RIPR in *P. vivax* erythrocyte invasion and parasite development (Knuepfer et al., 2019).

The polymorphism of malaria proteins involved in parasite invasion is a concern for vaccine development, as it may contribute to immune evasion. In this research, we successfully sequenced the *ripr* gene of *P. vivax*-infected blood samples obtained from patients in southern Mexico (SM), which, along with others from a public database, were analysed to understand the molecular diversity and evolutionary connections between parasites from this region and other geographical locations.

In this study, we investigated the genetic diversity, population structure and evolutionary pressures acting on the *pvripr* gene in *P. vivax* parasites from SM and compared them with parasites from other geographical regions. We hypothesized that, despite the essential role of RIPR in erythrocyte invasion, PvRIPR would exhibit signatures of site-specific positive selection driven by host immune pressure, particularly at surface-exposed residues and predicted B-cell epitopes, while remaining globally constrained at the sequence level. To test this hypothesis, we combined population genetic analyses, neutrality and selection tests, haplotype and phylogenetic approaches and *in silico* epitope mapping across global PvRIPR sequences.

## Materials and methods

All the samples analysed were obtained from adults (over 18 years of age) who signed a written consent under project approval CI1042 National Institute of Public Health – Mexico.

### *P. vivax* samples

The samples were obtained from different symptomatic patients between 2000 and 2015 from the Pacific side of Southernmost Chiapas, Mexico.

*P. falciparum* was eliminated in this region in the 1990s, leaving *P. vivax* as the unique species causing malaria. Most *P. vivax* symptomatic patients in this region experience an uncomplicated disease and seek diagnosis (Gonzalez-Ceron et al., 2016). *P. vivax* was diagnosed via microscopy using a Giemsa-stained thick smear. Capillary blood samples from finger pricks were used to saturate a circle of 2 cm marked on a filter paper Whatman #2. Samples were stored at 4 °C until further analysis.

### DNA extraction, PCR amplification and sequencing of *ripr* gene

From 7 samples, genomic DNA was extracted using the GeneJET Whole Blood Genomic DNA Purification Mini Kit (Thermo Fisher Scientific, Asheville, NC 28803, USA). To verify *P. vivax* infection, species-specific oligonucleotides were used (Supplementary Table 1) (Rubio et al., 1999). Six primers were designed to amplify the complete 3225 bp *pvripr* gene sequence (Supplementary Table 1). In order to avoid primer bias, *in silico* evaluation across global variants was carried out.

Because clinical samples infected with *P. vivax* typically contain low parasite densities, amplification of the full *pvripr* gene (∼3 kb) required a nested PCR strategy to increase template yield for sequencing. An initial external PCR was designed to amplify the entire coding region (3225 bp), followed by internal PCR reactions generating 2 overlapping fragments corresponding to the 5′ (1990 bp) and 3′ (1450 bp) portions of the gene (Supplementary Figure 1). Each overlapping fragment was sequenced using multiple internal sequencing primers, yielding individual Sanger reads of approximately 700–900 bp per reaction, which provided robust overlapping coverage across the full amplicon and enabled reliable assembly of the complete coding sequence (Supplementary Table 1).

The external PCR was performed using primers RIPR-For-ext and RIPR-Rev-ext with the high-fidelity Platinum SuperFi II DNA polymerase (Invitrogen) in a final volume of 20 µL. The reaction used 200 ng of the whole genome and was run in a MyGo Pro thermal cycler (Biocompare, Inc., Kansas City, MO 66103, USA). The PCR protocol included a denaturation step at 94 °C for 5 min, followed by 40 cycles, each at 94 °C for 45 s, 61 °C for 45 s and 72 °C for 60 s, and a final extension at 72 °C for 10 min. Afterwards, a nested PCR was performed using 0.1 µL of the previous PCR product (Green and Sambrook, 2019). The 5′ fragment was amplified using primers RIPR-For-Int and RIPR-Int-Up, and the 3′ fragment using RIPR-Int-Down and RIPR-Rev-Int. Nested PCR conditions consisted of 40 cycles of 94 °C for 30 s, 60 °C for 30 s and 72 °C for 30 s, with a final extension at 72 °C for 10 min. PCR products were verified on 1% agarose gels and visualized using a G:BOX F3 transilluminator (Syngene) (Supplementary Figure 1).

PCR products were purified and Sanger-sequenced in both directions using an ABI PRISM^®^ 3500 Genetic Analyzer (Applied Biosystems, Carlsbad, CA, USA) at the Central Research Services of the University of Almeria (Spain). Electropherograms were inspected and manually edited in Chromas v2.6.6 (Technelysium Pty Ltd) to trim low-quality regions and verify base calls. Sequences were curated to remove duplicates, detect artefacts and resolve annotation inconsistencies and coverage gaps. High-quality consensus sequences were generated and aligned with ClustalW in BioEdit v7.0.5.3 using the Sal-1 reference sequence (PVX_095055) (Thompson et al., 1994). No double peaks were detected in any electropherogram, suggesting the presence of only 1 genotype per isolate. This finding aligns with the low proportion of mixed-genotype infections reported in SM using various markers, e.g., microsatellites *pvama1* (Flores-Alanis et al., 2017) and *pvcyrpa* (González-Cerón et al., 2021). The sequences were deposited in the GenBank database, identified with the accession numbers OR569775–OR569781.

### Genetic analysis

The Sal-1 *ripr* sequence (PVX_095055) was used as a reference. For comparisons, homologous sequences from different geographical regions, regardless of their transmission intensity, were retrieved from PlasmoDB (https://plasmodb.org/plasmo/) using the Sal-1 gene identifier.

All the sequences included in the analysis and *P. vivax* populations were grouped per country or per region. The number of haplotypes (H), the nucleotide (π) and genetic diversity (θ), haplotype diversity, and the minimal number of recombination events (Rm) were calculated in DnaSP. The presence of breakpoints likely by recombination was also analysed using GARD (Genetic Algorithm for Recombination Detection), a program that uses phylogenetic incongruence among segments of a sequence alignment to detect the best-fit number and location of recombination breakpoints. Analyses were run on DataMonkey server (www.datamonkey.org), using default parameters (Kosakovsky Pond et al., 2006).

Neutrality tests (Tajima’s D, Fu and Li’s D* and F*) were used to evaluate whether the frequency spectrum of segregating sites deviated from neutral expectations at the population level, based on comparisons between nucleotide diversity (π) and genetic diversity (θ). These tests are sensitive to deviations from neutrality caused by both demographic processes and selection, but they do not distinguish between synonymous and nonsynonymous substitutions. Slide window analysis for the nucleotide diversity and Tajima’s D was run in DnaSP v6 (Rozas et al., 2017). To analyse the genetic relationships among Mexican isolates, a maximum likelihood phylogenetic tree using *pvripr* sequences from Mexican parasites was constructed using the best-fitting nucleotide substitution model by the Bayesian Information Criterion as Hasegawa–Kishino–Yano model (HKY+I), and bootstrap method with 1000 replications in MEGA v11 (Tamura et al., 2013). Only nodes with >70% bootstrap support were considered reliable. Also, haplotype networks were constructed for inferring and visualizing the genealogical relationships using PopART v1.7 (Bandelt et al., 1999).

Selective pressure acting on the *pvripr* coding region was assessed by comparing the rates of nonsynonymous (*dN*) and synonymous (*dS*) substitution using a *Z* test of selection using the alternative hypothesis: *dN* > *dS*. This test evaluates whether amino acid-changing mutations versus synonymous changes occur in excess relative to neutral expectations and is therefore indicative of selection acting at the protein level. A *Z* test of selection was run using a bootstrap method with 1000 replications in MEGA v11 (Tamura et al., 2013). The genetic differentiation among *P. vivax* populations was evaluated by *F*_ST_ index using DnaSP v6. This was based on the complete nucleotide alignment of the *pvripr* coding region, taking into account haplotype frequencies and nucleotide differences between populations.

To detect codons under positive selection across *pvripr* coding gene, the Single-Likelihood Ancestor Counting (SLAC Analysis) (Chang, 1999) was run on the Datamonkey server.

For the meticulous examination and visualization of disparities between synonymous (*dS*) and non-synonymous (*dN*) mutation rates within genetic sequences, SLAC, a method employed to pinpoint codon sites susceptible to positive, neutral or purifying selection, produces a graphical representation known as the SLAC Site Graph, encapsulating the outcomes of this analytical process (Pond and Frost, 2005; Kosakovsky et al., 2007). The X-axis of the graph denotes the ‘codon site’. The Y-axis encapsulates the variance between non-synonymous (*dN*) and synonymous (*dS*) mutation rates, expressed as *dN–dS*. This disparity acts as a gauge for positive or purifying selection at each codon site (Muyiwa, 2015).

To evaluate patterns of selection along the *pvripr* coding region, we employed codon-based approaches implemented in the HyPhy package through the Datamonkey web server (https://www.datamonkey.org). Site-specific estimates of nonsynonymous and synonymous substitution rates (*dN* and *dS*) were first obtained using the SLAC method, which identifies codons with statistically significant deviations of *dN–dS* (*P* < 0.10).

To validate the presence of pervasive selection, we additionally applied the Fast, Unconstrained Bayesian AppRoximation (FUBAR) method. Codons with posterior probability ≥0.90 of *dN* > *dS* were interpreted as being under positive selection, whereas codons with posterior probability ≥0.90 of *dN* < d*S* were interpreted as being under negative (purifying) selection. Both analyses were performed using the maximum-likelihood phylogeny inferred under the HKY+I substitution model (Supplementary Figure 2). Codons were regarded as showing robust evidence of positive selection when they were significant in SLAC and simultaneously exceeded the FUBAR posterior-probability threshold for *dN* > *dS*, whereas robust purifying selection was inferred when codons showed negative *dN*–*dS* values in SLAC and posterior probability ≥0.90 for *dN* < *dS* in FUBAR.

### Potential residues participating in B-cell epitopes and with probability to be exposed on the protein surface in polymorphic residues in PvRIPR

The physicochemical properties of PvRIPR were first inferred from the Sal-1 reference sequence (PVX_095055). The predicted molecular weight (121 KDa) and theoretical isoelectric point (pI = 7.68/slightly basic) were obtained using ProtParam (ExPASy server; https://web.expasy.org/cgi-bin/protparam/), and the presence of a signal peptide of 21 amino acids was evaluated with SignalP-6.0 (https://services.healthtech.dtu.dk/services.SignalP-6.0). As reported for other *Plasmodium* RIPR orthologues, PvRIPR contains 10 EGF-like domains distributed along the N- and C-terminal regions, 2 on the N-terminal peptide and 6 on the C-terminal side.

There is evidence that this protein is processed into 2 segments of similar molecular weight, and the cleavage site is suggested between the second and third EGF-like domains (Supplementary Table 2) (Chen et al., 2011; Lauron et al., 2015; Knuepfer et al., 2019). PvRIPR amino acid polymorphism and its frequency were compared between samples from SM and other geographical sites.

To investigate whether polymorphic residues might be part of B-cell epitopes, the Sal-1 PvRIPR sequence was analysed using BepiPred Linear Epitope Prediction 2.0 (BepiPred 2.0), implemented in the Immune Epitope Database (IEDB; https://www.iedb.org). BepiPred 2.0 combines a hidden Markov model with amino acid propensity scales to predict linear B-cell epitopes. For each residue, the BepiPred Linear Epitope Prediction score (BLEP score) was retrieved. A threshold of 0.50 was applied, representing a balance between sensitivity and specificity; residues with BLEP >0.50 were considered likely to belong to a linear B-cell epitope.

Surface accessibility was assessed using the Emini surface accessibility prediction (ESAP) method, also available through IEDB. For each residue, an ESAP score was obtained, calculated over a hexapeptide window centred on the residue of interest. Hexapeptides with ESAP values >1.0 were interpreted as having an increased probability of being exposed to the protein surface.

Polymorphic amino acid positions identified in the population analyses were mapped onto the Sal-1 PvRIPR sequence, and their corresponding BLEP and ESAP scores were retrieved. Attention was given to polymorphic residues located within EGF-like domains and/or within predicted B-cell epitopes (BLEP >0.50) and surface-exposed regions (ESAP >1.0).

## Results

### *pvripr* polymorphism of parasites from SM

**Figure 1.**
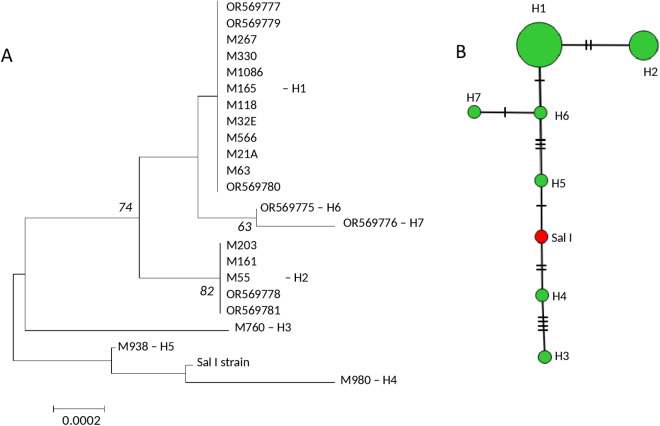
Genetic relationships among 22 *Plasmodium vivax* isolates from southern Mexico based on the *pvripr* gene sequence. (A) ML tree; (B) haplotype network. OR isolates correspond to sequences generated in this study, whereas M isolates were retrieved from PlasmoDB. The ML tree topology reveals 2 main clusters supported by 74% bootstraps. One cluster includes the *Sal I* strain and haplotypes H5, H4 and H3, each represented by a single isolate, whereas the second cluster comprises a larger group that includes the 2 most frequent haplotypes, H1 and H2. The haplotype network shows that the haplotypes were separated by 1–4 mutational steps within them and from 1 to 13 mutational steps between them. *Sal I* haplotype was located in the middle of the 2 genetic groups defined by the ML tree. Figure 1 long description.

Complete *pvripr* gene sequences were obtained from 7 Mexican isolates and were combined with 15 sequences retrieved from the PlasmoDB database (all collected between 2001 and 2015), resulting in a dataset of 22 sequences encompassing 3222 bp. Supplementary Table 2 details the mutations identified among the 9 polymorphic codons: 19, 53, 81, 339, 357, 371, 373, 616 and 634. This group had nucleotide diversity of π = 0.0007, which resolved 7 haplotypes, and haplotype diversity of 0.67. Haplotype H1 included 12 isolates, H2 5 isolates, while 5 haplotypes were unique. The ML tree revealed 2 genetic groups separated by 74% bootstrap support, 1 with 3 isolates (h3, h4 and h5) from the other with 19 isolates, and these were split into 2 further genetic groups by an internal branch with 82% bootstrap support. While the haplotype network showed one more frequent haplotype, and the haplotypes were separated by 1–4 mutational steps within them and from 1 to 13 mutational steps between them. Sal I haplotype was in the middle of the 2 main genetic groups defined by the ML tree (Figure 1; Supplementary Figure 3).

### Global comparison of genetic parameters and neutrality test analysis

Sequences were grouped by country (Mexico, Colombia, Peru, Thailand and China) with an additional East Asia and Southeast Asia (EA-SEA) group encompassing parasites from China, Thailand, Cambodia (*n* = 3) and Papua New Guinea (*n* = 5). Parasites from Latin America were also analysed as a single group. Sampling periods were 2001–2015 for Mexico and 2010–2012 for other regions. Latin American parasites exhibited fewer mutations compared to EA-SEA parasites, despite showing similar haplotype numbers. Notably, Mexican parasites displayed the lowest nucleotide diversity compared to other regions (*π* = 0.0011–0.0017, 10–12 haplotypes, Hd > 0.9; Supplementary Table 3). Sliding window analysis revealed differences between nucleotide diversity (π) patterns between Latin American and EA-SEA populations along the *ripr* gene (Supplementary Figure 4). While Thai and Chinese parasites displayed π values across the entire sequence, American parasites showed minimal polymorphism beyond the ∼2000 nucleotide mark.

The minimum number of recombination events (Rm) varied among populations (Table 1), from 2 in SM to 5 in Peru, and in EA-SEA was slightly higher. Evidence of recombination was detected in all sequence alignments. GARD suggested potential breakpoints from 9 in Mexican sequences to 19 in those from China. In all populations except that from Thailand, 2 breakpoints were inferred, and the gene location varied among populations, and the position of recombination breakpoints along the *pvripr* gene varied among populations.

**Table 1.** Neutrality and selection tests using *P. vivax ripr* gene from different geographical origins Table 1 long description.

		Neutrality tests			*Z* test of selection			
Geographical origin	*N*	Tajima's D	Fu and Li D*	Fu and Li F*	S	NS	dN–Ds	*P-*value
Southern Mexico	22	−0.6710	−0.06183	−0.27866	0	10	2.79	0.01
Colombia	23	0.66246	0.51481	0.64987	1	10	1.75	0.04
Peru	17	0.69773	0.47588	0.62129	1	13	2.91	0.00
Thailand	11	−0.7944	−0.83704	−0.93611	6	13	0.0	1.0
China	19	−0.25363	0.01050	−0.07695	6	14	0.17	0.43

S, synonymous; NS, non-synonymous. Neutrality tests were run in DnaSP v6, and *Z* test was run in MEGA v11.

Tajima’s D, Fu and Li’s D* and F* statistics were not significant for any population, indicating no evident deviation from neutrality (Supplementary Figure 5). The rate of nonsynonymous substitutions exceeded that of synonymous substitutions, and a Z test of selection (HA: dN > dS) yielded significant positive values (Table 1). In Mexican parasites, all mutations were nonsynonymous, and these predominated in parasites from Peru and Colombia. Overall, these results provide evidence of selective pressure acting on the amino acid residues in the PvRIPR protein.

### SLAC and FUBAR analysis of P. vivax populations using *ripr* gene

**Figure 2.**
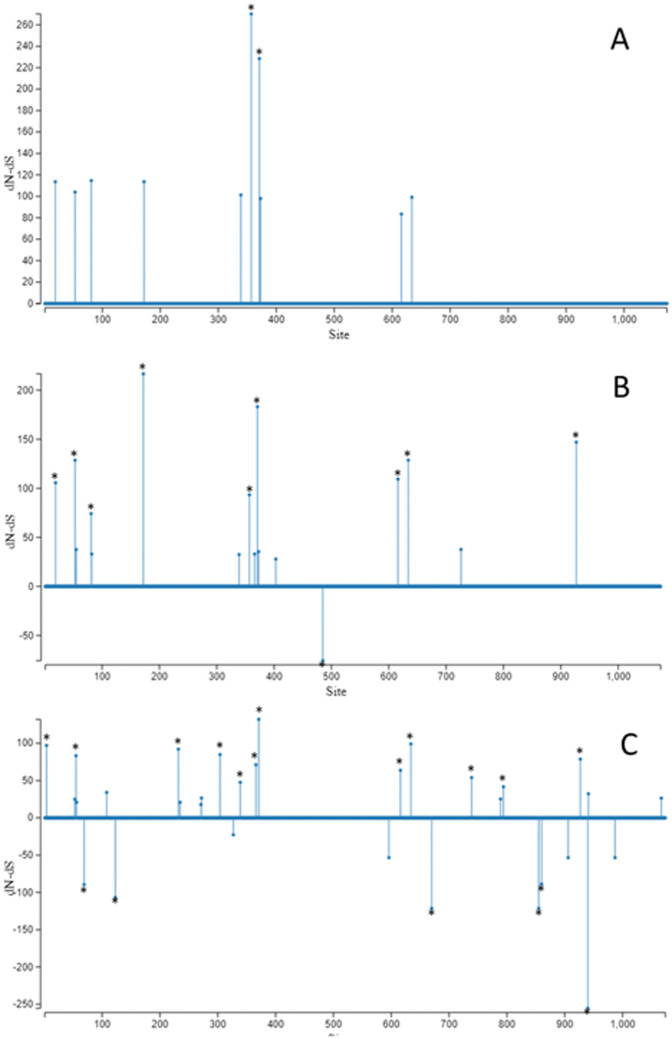
Codon-level selection analyses of the *pvripr* gene. SLAC profiles show site-wise *dN*–*dS* values (positive and negative) across the coding sequence in parasites from (A) Mexico (*n* = 22), (B) Latin America (*n* = 62) and (C) EA–SEA (*n* = 38). Bars represent dN–dS estimate for each codon, and asterisks indicate sites with statistically significant SLAC values (*P*<0.10). Codons additionally supported by FUBAR (posterior probability ≥0.90 for positive or purifying selection) are described in the main text. Figure 2 long description.

To determine whether the excess of nonsynonymous substitutions was driven by selection acting on specific regions of the gene, codon-based methods were applied to identify individual sites under positive or purifying selection. SLAC revealed substantial heterogeneity in dN–dS values across the *pvripr* gene in all populations analysed (Figure 2). In parasites from SM, SLAC identified 10 codons with positive dN–dS values; however, only codons 357 and 371 were also supported by FUBAR (posterior probability ≥0.90 for *dN* > *dS*) and were therefore considered to be under robust positive selection. In the broader Latin American dataset, 9 codons exceeded the FUBAR posterior-probability threshold (19, 53, 81, 172, 357, 371, 616, 634 and 927), most of them located in PvRIPR N-terminal segment.

The EA-SEA population displayed the highest number of selected sites, with 12 codons showing evidence of positive selection (4, 55, 232, 304, 339, 366, 371, 616, 634, 739, 794 and 927) and 6 codons exhibiting evidence of significant purifying selection (69, 123, 670, 855, 860 and 940). In this group, several codons with negative dN–dS values identified by SLAC also showed high posterior probability in FUBAR (dN < dS, posterior probability ≥0.90), supporting the presence of strong purifying selection at these sites.

In the SLAC profiles, asterisks denote codons with statistically significant SLAC values (*P* < 0.10), whereas the subset of positively and negatively selected sites was corroborated by FUBAR (Figure 2).

### Haplotype sharing and genetic relationships

One mutation at codon 373 (TTG/Leu to TGG/Trp) was unique to a single isolate (H7) from Mexico. The mutation at codon 19 was shared with Colombian parasites, while the mutation at codon 81 was shared with parasites from Peru. The remaining 7 mutations were shared with parasites from other geographic regions. Despite this, *P. vivax ripr* haplotypes from Mexico were predominantly unique, with only the most common haplotype shared with Colombian parasites. Other geographic regions also exhibited exclusive haplotypes with a lack of high-frequency haplotypes.

The lowest *F*_ST_ index was estimated between parasites from Mexico and Colombia (0.156) and was similar to that between Colombia and Peru, or between Thailand and China. In contrast, the higher differentiation values were detected between Mexican or Colombian and Asian parasites. *F*_ST_ index between South American and Asian parasites was 0.301, like that detected between Peruvian and all parasite populations (Table 2).

**Table 2.** *F*_ST_ indexes between *P. vivax* populations from different geographic locations using *ripr* gene Table 2 long description.

	Mexico	Colombia	Peru	Thailand
Colombia	0.156			
Peru	0.316	0.181		
Thailand	0.561	0.487	0.367	
China	0.411	0.374	0.300	0.164

### Polymorphic residues potentially participating in the B-cell epitopes

Linear B-cell epitope and surface-accessibility predictions were performed using the methods described in the Materials and Methods section. Among the 19 amino acid changes observed in more than 1 isolate, 12 involved residues with a charged side chain, and their frequencies varied across geographical regions (Supplementary Table 2). The substitutions G19D, E53R, D357E, E371A, K616N and K634E showed moderate-to-high frequencies in SM (22–95%) and were also present in other regions, except for G19D, which was restricted to isolates from Mexico and Colombia. Three low-frequency substitutions (A81E, L172I and E339R) were detected at <10%, with A81E additionally reported in Peru, whereas E373A was exclusive to SM.

The PvRIPR protein is cysteine-rich (8.6%), and all cysteine residues were conserved across isolates. However, some polymorphisms resulted in marked physicochemical changes. For example, the substitution G19D replaces a small, neutral residue with a larger, negatively charged amino acid, whereas in a subset of Chinese isolates, T272P substitutes a polar residue with a hydrophobic and structurally restrictive proline. Two substitutions, located within the predicted signal peptide (positions 4 and 19), were detected in Asian and Latin American parasites, respectively.

Nine polymorphic residues occurred within EGF-like domains, and 7 of these involved charged amino acids. Eleven of the polymorphic sites exhibited BLEP scores >0.5 and ESAP scores >1.0, 6 of which were in EGF-like domains (Table 3), suggesting that genetic variation in PvRIPR may influence both surface exposure and the likelihood of contributing to linear B-cell epitopes.

**Table 3.** Comparison of the protein polymorphism of PvRIPR in parasites from different geographical locations and its potential to participate in B-cell epitopes Table 3 long description.

Amino acid position, and change	Frequency of amino acid changes at *P. vivax* RIPR protein (%):						BLEP 2.0 AA-score (peptide*)	ESAP Scores, hexapeptides
	Mexico (*n* = 22)	Colombia (*n* = 23)	Peru (*n* = 17)	Thailand (11)	China (19)	EA-SEA (38*^,^**)		
4, R→W^1^	0	0	0	9.1	5.3	7.9*	–	0.12, KCRVCI
19, G→D^1^	63.6	34.8	0	0	0	0	0.49	2.59, RRGRAN
53, E→R^1^	22.7	52.2	26.1	18.2	10.5	21.1*^,^**	0.54 (44−66)	3.60, KKERVD^6^
55, V→L	0	0	11.8	81.8	31.6	50.0*^,^**	0.56 (44−66)	2.03, ERVDYT^6^
81, A→E^1^	0.091	0	0.318	0	0	0	0.55 (76−95)	1.22, SEAEIK
172, L→I	9.1	4.3	58.8	100	100	100*^,^**	0.42	0.69, EELFFQ
232, K→N^1^	0	0	0	0	21.1	13.2**	0.58 (222−270)	1.36, VKKGNS
272, T→P^2^	0	0	0	0	36.8	–	0.48	0.91, RMTTCR
^5^304, F→Y	0	0	0	27.3	10.5	–	0.51 (304−307)	0.80, NQFCDE
^5^339, E→R^1^	4.5	52.2	35.3	100	52.6	73.7*^,^**	0.49	0.75, CPEDAT
^5^357, D→E	81.8	73.9	64.7	100	100	100*^,^**	0.51 (357−373)	1.29, RCDEGK
^5^366, E→K^1^	0	0	23.5	100	47.4	73.7*^,^**	0.54 (357−373)	1.61, KNECYN
371, E→A^1^	90.9	95.7	52.9	45.5	78.9	71.1*^,^**	0.55 (357−373)	1.45, NMEELE
403, I→V	0	13.0	0	0	0	0	0.54 (357−477)	0.75, EHIYMH
^5^616, K→N^1^	95.5	87.0	64.7	72.7	89.5	81.6*^,^**	0.55 (613−640)	3.36, SKKNHS^9^
^5^634, K→E^1^	86.4	43.5	11.8	72.7	94.7	89.5	0.52 (613−640)	0.44, CGKNQI
^5^789, E→K^1^	0	0	0	0	15.8	–	0.46	1.05, CKENLK
^5^794, N→D^1^	0	0	0	90.9	78.9	73.7**	0.55 (791−825)	4.67, KRNSNN^8^
^5^927, R→H^1^	0	13.0	11.8	18.2	0	15.8*^,^**	0.55 (926−985)	3.17, NYRPRG^7^

Table values indicate the frequency (%) of each amino acid substitution in the PvRIPR protein relative to the Sal-1 reference sequence for each geographic region. PvRIPR has 1074 amino acids, including a signal peptide. BLEP, Bepipred Linear Epitope prediction, ESAP, Emini surface accessibility prediction. Includes isolates from PNG* (5 isolates) and/or Cambodia** (3 isolates). 373, L→W was exclusive to 1 isolate from Mexico. Amino acid change at codons 82, E→D and 726, L→V each was exclusive to 1 isolate from Peru, respectively; and changes at codons 108, L→P and 739, Q→K each in 1 isolate from Thailand, respectively. Mutations at 235, S→N and 327, L→F both were present in 1 isolate in PNG. BLEP scores min 0.141 and a maximal value of 0.653. EA-SEA, East Asia-Southeast Asia. As a reference, Sal I strain was used. ^5^Indicate residues positioned in EGF-like domains. Ten Hexapeptides comprising residues 43–58^6^ and 9 hexapeptides containing residues 923–936^7^ had ESAP scores of 2.0–7.8 and 2.12–5.54, respectively. Six hexapeptides containing residues 788–798 had scores of 2.4–4.7^8^; hexapeptides with residues 613–616 had scores of 2.66–4.34^9^, and those with residues 355–376 had scores of 1.0–2.4. EGF-like domains comprise residues 293–368 and 583–930.

## Discussion

The P. vivax RH5-interacting putative protein (PvRIPR) exhibited lower genetic diversity in the Southern Mexican parasite population compared with other geographic regions. An excess of nonsynonymous substitutions and signals of positive selection were detected in pvripr from Mexico and South America, as indicated by Z-test values. At a global scale, pvripr haplotypes were predominantly low-frequency and geographically restricted.

The observed complexity of haplotype networks may be partly explained by recombination events, as suggested by the minimal number of recombination signals detected by the GARD algorithm, as well as by uncertainty in mutational ordering, homoplasy, and limited phylogenetic resolution associated with datasets dominated by rare or unique haplotypes. In addition, several amino acid substitutions identified across Mexican and other populations were predicted to fall within putative B-cell epitopes, suggesting possible immune-driven selection.

At the population structure level, P. vivax from Mexico showed the lowest FST values when compared with Colombian isolates and the highest differentiation relative to Asian parasites. Overall, many of the amino acid substitutions identified in Mexican and other populations were located in residues predicted to participate in B-cell epitopes, further supporting the role of immune-mediated selection on PvRIPR.

Although the specific role of PvRIPR in *P. vivax* reticulocyte invasion remains unclear, substantial evidence from other *Plasmodium* species supports a conserved role for RIPR in erythrocyte interaction and invasion-related processes. In *P. falciparum*, RIPR is a core component of the RH5 invasion complex and has also been shown to independently bind human erythrocytes (Chen et al., 2011; Nagaoka et al., 2020). Functional studies in *P. falciparum* and *P. knowlesi* demonstrate that disruption of RIPR expression or antibody-mediated targeting significantly impairs parasite invasion and growth. On the other hand, an anti-basigin polyclonal serum demonstrated a broad spectrum of growth inhibition (0–80%) across various *P. vivax* isolates (Knuepfer et al., 2019). This pattern is similar to the effect of polyclonal antibodies against PfRIPR, which inhibited merozoite adhesion and reduced parasite growth to varying degrees in different *P. falciparum* strains (Chen et al., 2011).

It should be noted that neutrality tests based on the site-frequency spectrum, such as Tajima’s D and Fu and Li’s statistics, are sensitive to both demographic history and selection, but do not allow discrimination between these processes. Overall, population-level neutrality tests (Tajima’s D, Fu and Li’s D* and F*) did not show significant deviations from neutral expectations in any region, indicating that *pvripr* evolution is broadly consistent with neutrality when assessed using the frequency spectrum of segregating sites. However, because these tests do not distinguish between synonymous and nonsynonymous mutations, they do not capture selective pressures acting specifically on the protein sequence. In contrast, dN–dS analyses revealed a significant excess of nonsynonymous substitutions in Mexican and South American parasites, supported by positive *Z* test values; notably, all mutations detected in Mexican isolates were nonsynonymous, with similar patterns in parasites from Peru and Colombia. Codon-based approaches further showed that positive selection is not uniform across the gene but restricted to specific sites, several of which are located in predicted surface-exposed regions and B-cell epitopes. Together, these results indicate that while *pvripr* shows no strong departure from neutrality at the population level, the PvRIPR protein is subject to site-specific positive selection, likely driven by host immune pressure. In this context, the low overall genetic diversity observed in Mexican and South American populations suggests that PvRIPR is functionally constrained yet experiences localized immune-driven selection, a pattern consistent with essential proteins that remain targets of host immunity and that justifies further functional and immunological characterization of PvRIPR.

Proteins forming the RH5 complex (RH5, CYRPA and RIPR) in *P. falciparum* from African regions exhibit low polymorphism (Ntege et al., 2016; Ndwiga et al., 2021; Waweru et al., 2023). The *pvripr* gene in Ugandan parasites shows an excess of non-synonymous mutations and a historical population expansion, similar to what was observed in Kenyan parasites (Ntege et al., 2016; Ndwiga et al., 2021). Unlike Ugandan parasites, *P. falciparum* from Kenya has amino acid substitutions primarily in the N-terminal peptide, and these substitutions do not involve charged amino acids. Additionally, some amino acid changes involved gaining or losing cysteines (Ndwiga et al., 2021).

Limited immune responses against the RH5 complex have been observed in *P. falciparum* (Waweru et al., 2023). Healer et al. (2019) demonstrated that neutralizing monoclonal antibodies against PfRIPR inhibit merozoite invasion via a mechanism distinct from RH5 complex blockade. Moreover, a polyclonal antibody targeting conserved EGF-like domains 5–9 of PfRIPR (residues 720–934) achieved 50–70% inhibition of merozoite invasion in a concentration-dependent manner (Wong et al., 2019). Amino acid substitutions in PfRIPR have been identified outside of EGF-like domains. Similarly, in *P. vivax*, 13 of 26 global amino acid substitutions were located outside EGF-like domains in the N-terminal region. Additionally, 7 polymorphic residues were found in the first and second N-terminal domains. Six amino acid changes were distributed across C-terminal domains (residues 616–927), potentially contributing to linear or conformational B-cell epitopes.

A previous study comparing PvCYRPA (encoded by 2 exons) to PvRIPR in similar parasite populations revealed a 10-fold higher nucleotide diversity in PvCYRPA (González-Cerón et al., 2021). This suggests that PvRIPR may have less exposure to the immune system than PvCYRPA. Exon 1 of PvCYRPA was suggested to be under balancing selection and involved in immune evasion.

*P. vivax* and *P. knowlesi* are closely related species lacking the RH5 gene (Knuepfer et al., 2019). While *P. falciparum* forms a complex with PTRAMP and CSS proteins, *P. knowlesi* utilizes a complex involving PTRAMP and CSS, and anti-PkRIPR antibodies significantly reduce parasite density (Knuepfer et al., 2019). Whether *P. vivax* forms a similar complex and if the observed amino acid polymorphism in PvRIPR from various locations contributes to immune evasion requires further experimental investigation.

## Supporting information

10.1017/S0031182026102157.sm001 Cebrian-Carmona et al. supplementary material Cebrian-Carmona et al. supplementary material

## Supplementary material

The supplementary material for this article can be found at https://doi.org/10.1017/S0031182026102157.

## Figure 1 Long description

The image consists of two parts labeled A and B. Part A is a Maximum Likelihood (ML) tree depicting genetic relationships among Plasmodium vivax isolates. The tree is oriented vertically, with branches extending downward. The tree begins with a main branch that splits into two clusters. The first cluster, supported by 74 percent bootstrap, includes isolates OR569777, M267, M330, M1068, M118, M32E, M556, M21A, M63 and OR569780, all associated with haplotype H1. The second cluster, supported by 82 percent bootstrap, includes isolates M203, M161, M55, OR569778, OR569781 and M760, associated with haplotypes H6 and H7. The Sal I strain is positioned between haplotypes H5 and H4, with M938 linked to H5 and M980 linked to H4. A scale bar indicating 0.0002 is present at the bottom. Part B is a haplotype network showing relationships among haplotypes H1 to H7. The network is vertically oriented, with circles representing haplotypes. H1 and H2 are the largest and most frequent, located at the top. H3, H4, H5, H6 and H7 are smaller and connected by lines indicating mutational steps. The Sal I haplotype is marked in red and positioned centrally between the two main genetic groups defined by the ML tree. Lines between haplotypes represent 1 to 4 mutational steps within groups and 1 to 13 steps between groups.

Navigate back to Figure 1.

## Figure 2 Long description

The image A showing a vertical bar graph labeled A. The horizontal axis label is Site, with tick labels 100, 200, 300, 400, 500, 600, 700, 800, 900 and 1,000. The vertical axis label is dN minus dS, with tick labels 0, 20, 40, 60, 80, 100, 120, 140, 160, 180, 200, 220, 240 and 260. Vertical bars rise from the baseline at 0 at multiple sites, including bars near Site 0, near Site 50, near Site 100, near Site 200, a cluster between Site 300 and Site 400 with two tallest bars reaching near 260 and near 240 and bars near Site 650 reaching near 100. The image B showing a vertical bar graph labeled B. The horizontal axis label is Site, with tick labels 100, 200, 300, 400, 500, 600, 700, 800, 900 and 1,000. The vertical axis label is dN minus dS, with tick labels negative 50, 0, 50, 100, 150 and 200. Several vertical bars rise above 0 near Site 0 to 120, including one near Site 200 reaching slightly above 200. A cluster of bars appears between Site 300 and Site 420, including one reaching near 180. A long vertical bar extends below 0 near Site 500 to about negative 50. Additional bars appear near Site 600 to 650 reaching around 120 to 140, a smaller bar near Site 700 around 50 and a bar near Site 900 around 150. The image C showing a vertical bar graph labeled C. The horizontal axis label is Site, with tick labels 100, 200, 300, 400, 500, 600, 700, 800, 900 and 1,000. The vertical axis label is dN minus dS, with tick labels negative 250, negative 200, negative 150, negative 100, negative 50, 0, 50 and 100. Bars appear both above and below 0. Near Site 0 to 120, bars include one above 0 near 100 and two below 0 near negative 100. Between Site 200 and Site 420, multiple bars rise above 0 up to about 100, with one bar near Site 400 slightly above 100. Near Site 600 to 650, one bar rises above 0 near 100 and one drops below 0 near negative 150. Between Site 700 and Site 850, several bars rise above 0 around 40 to 80. Near Site 850 to 950, multiple bars drop below 0, including one near Site 900 reaching about negative 250 and other bars near negative 100 to negative 150. Near Site 950 to 1,000, bars appear above 0 around 40 to 80 and below 0 around negative 50 to negative 100.

Navigate back to Figure 2.

## Table 1 Long description

The table reports neutrality statistics and a selection test for ripr gene samples from five regions, along with sample size and counts of synonymous and non-synonymous changes. Southern Mexico has 22 samples, no synonymous changes, 10 non-synonymous changes, and a significant selection test with a high positive difference between non-synonymous and synonymous rates and a low p value. Peru shows the strongest signal: 17 samples, 1 synonymous and 13 non-synonymous changes, the largest positive rate difference, and a p value reported as zero. Colombia also indicates selection but more moderately, with 23 samples, 1 synonymous and 10 non-synonymous changes, and a p value of 0.04. Thailand differs from the Americas: 11 samples, 6 synonymous and 13 non-synonymous changes, a zero rate difference, and a non-significant p value of 1.0. China is also non-significant, with 19 samples, 6 synonymous and 14 non-synonymous changes, a small positive rate difference, and a p value of 0.43. Neutrality statistics vary in sign across regions, so interpretation should rely primarily on the selection test results and consider that methods and software choices can affect these estimates.

Navigate back to Table 1.

## Table 2 Long description

The table reports pairwise FST index values comparing Plasmodium vivax populations from Mexico, Colombia, Peru, Thailand, and China using the ripr gene. Higher FST values indicate greater genetic differentiation between the two locations. The largest value is for Thailand compared with Mexico at 0.561, followed by Thailand compared with Colombia at 0.487 and China compared with Mexico at 0.411. The smallest value is for Thailand compared with China at 0.164, with other relatively low values including Colombia compared with Mexico at 0.156 and Peru compared with Colombia at 0.181. Peru shows moderate differentiation from Mexico at 0.316 and from Thailand at 0.367, and China is moderately differentiated from Peru at 0.300 and from Colombia at 0.374. Values are shown only once per pair in a lower-triangular layout, so blank cells do not indicate missing comparisons.

Navigate back to Table 2.

## Table 3 Long description

The table lists PvRIPR amino acid substitutions by position and reports how often each change occurs in parasite samples from Mexico, Colombia, Peru, Thailand, China, and a combined East Asia–Southeast Asia set. Several substitutions are fixed or nearly fixed in some Asian groups, including L to I at position 172 at 100 percent in Thailand, China, and the combined group, and D to E at position 357 at 100 percent in Thailand, China, and the combined group. Other changes are common but vary by region, such as E to A at position 371 ranging from 45.5 percent in Thailand to 95.7 percent in Colombia, and E to R at position 339 reaching 100 percent in Thailand but 4.5 percent in Mexico. Some substitutions are largely regional, for example G to D at position 19 is frequent in Mexico and Colombia but absent in Peru, Thailand, China, and the combined group, while V to L at position 55 is high in Thailand and moderate in China and the combined group but absent in Mexico and Colombia. The last two columns provide predicted B cell linear epitope scores and surface accessibility scores for nearby peptides, indicating that several polymorphic regions overlap peptides with moderate predicted epitope potential and varying accessibility. Interpretation should consider that sample sizes differ by region and some entries are missing for the combined group, so rare or single-isolate changes may not generalize.

Navigate back to Table 3.
