# Supplementary material for: Unravelling the genetic diversity of RIPR: a key invasion protein in Plasmodium vivax malaria
Source: Parasitology. 2026 May 26;153(6):851–60. doi: 10.1017/S0031182026102157 (PMC13420167; doi:10.1017/S0031182026102157)
Supplement: Cebrian-Carmona et al. supplementary material [file S0031182026102157sup001.docx]

**Supplementary Tables**

**Supplementary Table 1.** PCR primers used for the amplification of the *pvripr* gene

| Primer | Sequence (5´- 3´) | Position | Specificity | Reference |
| --- | --- | --- | --- | --- |
| *P. vivax* diagnosis: |  |  |  |  |
| Reverse primer *UNR* | GACGGTATCTGATCGTCTTC | --- | Universal | Rubio et al., 1999  Rubio et al., 1999  Rubio et al., 1999 |
| Forward primer *PLF* | AGTGTGTATCCAATCGAGTTTC | *---* | *Plasmodium* |  |
| Reverse primer *VIR* | AGGACTTCCAAGCCGAAGC | *---* | *P. vivax* |  |
| PvRIPR primers used for amplification and sequencing: | | | | |
| RIPR-For-ext | GTTGGAGAGGGCAACTGGCCAGTGG | *-99 // -74* | *P. vivax*  *P. vivax*  *P. vivax*  *P. vivax*  *P. vivax*  *P. vivax* | This study  This study  This study  This study  This study  This study |
| RIPR-Rev-ext | CAGGCACAGGCACACCGGCTAAGC | *3243 // 3219* |  |  |
| RIPR-For-int | TCCGCGCGGACAGAAAG*ATGAAGTGC | *-17 // 9* |  |  |
| RIPR-Rev-int | AGGCACACCGGCTAAGCCTCCTCC | *3236 // 3212* |  |  |
| RIPR-Int-Down | GTGTGGCCCTAATTCGTCCTGCTACG | *1769 // 1795* |  |  |
| RIPR-Int-Up | GCAGCTACCTCGTGCTTCGCAAATGG | *1881 // 1855* |  |  |

*ATG is the start codon of the *pvripr* gene, so the adenine is position 1

**Supplementary Table 2.** Nucleotide and amino acid polymorphism of *P. vivax* *ripr* from southern Mexico

| Codon | N | 19 | 53 | 81 | 172 | 339 | 357 | 371 | 373 | 616 | 634 |
| --- | --- | --- | --- | --- | --- | --- | --- | --- | --- | --- | --- |
| Amino acid |  | Gly | Glu | Ala | Leu | Glu | Asp | Glu | Leu | Lys | Lys |
|  |  | GGC | GAA | GCG | CTA | GAG | GAC | GAA | TTG | AAG | AAG |
| Haplotype |  |  |  |  |  |  |  |  |  |  |  |
| H1 | 12 | .A. | ... | ... | ... | ... | ..G | .C. | ... | ..C | G.. |
| H2 | 5 | ... | A.. | ... | ... | ... | ..G | .C. | ... | ..C | G.. |
| H3 | 1 | ... | ... | .A. | A.. | A.. | ..G | .C. | ... | ..C | ... |
| H4 | 1 | ... | ... | .A. | A.. | ... | ... | ... | ... | ... | ... |
| H5 | 1 | ... | ... | ... | ... | ... | ... | ... | .. | ..C | ... |
| H6 | 1 | .A. | ... | .. | ... | ... | .. | .C. | ... | ..C | G.. |
| H7 | 1 | .A. | ... | .. | ... | ... | .. | .C. | .G. | ..C | G.. |
| Amino acid change | | Asp | Lys | Glu | Ile | Lys | Glu | Ala | Trp | Asn | Glu |

Analysis of complete coding gene of 3222 bp and 1074 codons. DNA sequence from 7 isolates was obtained in this study and other 15 were retrieved from PlasmoDB. N, number of sequences. Amino acids are indicated in three letter codes. All mutations were non-synonymous. *Sal-1 sequence was used as reference (PVX_095055 and Sal-1 from PlasmoDB). Only one singleton was detected. EGF-like domains comprise 293-368 and 583-930 residues (in bold).

**Supplementary Table 3**. Comparison of the parameters of diversity of *P. vivax* *ripr* gene from southern Mexico and other geographic origins

| Parameters of  Diversity | Mexico | Colombia | Peru | Latin America |
| --- | --- | --- | --- | --- |
| N | 22 | 23 | 17 | 62 |
| SS | 10 | 11 | 14 | 18 |
| M | 10 | 11 | 14 | 18 |
| H | 7 | 12 | 11 | 29 |
| Hd ± SD | 0.671 ± 0.0088 | 0.921 ± 0.034 | 0.934 ± 0.039 | 0.932 ± 0.021 |
| Π | 0.00068±0.0002 | 0.00110±0.0001 | 0.00152±0.0001 | 0.0013±0.0001 |
| θw ± (SD) | 0.0009± (0.0004) | 0.0009 ± (0.0004) | 0.0013 ± (0.0006) | 0.0012 (0.0004) |
| Singletons | 1 | 2 | 3 | 4 |
| Rm | 2 | 3 | 5 | 6 |
|  | Thailand | China |  | EA-SEA |
| N | 11 | 19 |  | N=38 |
| SS | 19 | 20 |  | 29 |
| M | 19 | 20 |  | 30 |
| H | 10 | 11 |  | 27 |
| Hd ± SD | 0.982 ± 0.046 | 0.912 ± 0.046 |  | 0.973 ± 0.015 |
| Π | 0.00166±0.0003 | 0.0017±0.0002 |  | 0.0018±0.0002 |
| θw ± (SD) | 0.0020 ± (0.00089) | 0.0018 ± (0.0007) |  | 0.0021 ± (0.00073) |
| Singletons | 11 | 6 |  | 10 |
| Rm | 4 | 5 |  | 11 |

N, number of isolates; SS, segregating sites; M, mutations; H, haplotypes; Hd, haplotype diversity; π denotes nucleotide diversity; θw represents Watterson’s estimator of genetic diversity; SD indicates standard deviation; EA, East Asia; SEA, Southeast Asia; Rm, minimal number of recombination events. EA-SEA included sequences from Thailand, China, 5 from PNG and 3 from Cambodia.

**Supplementary Figures**


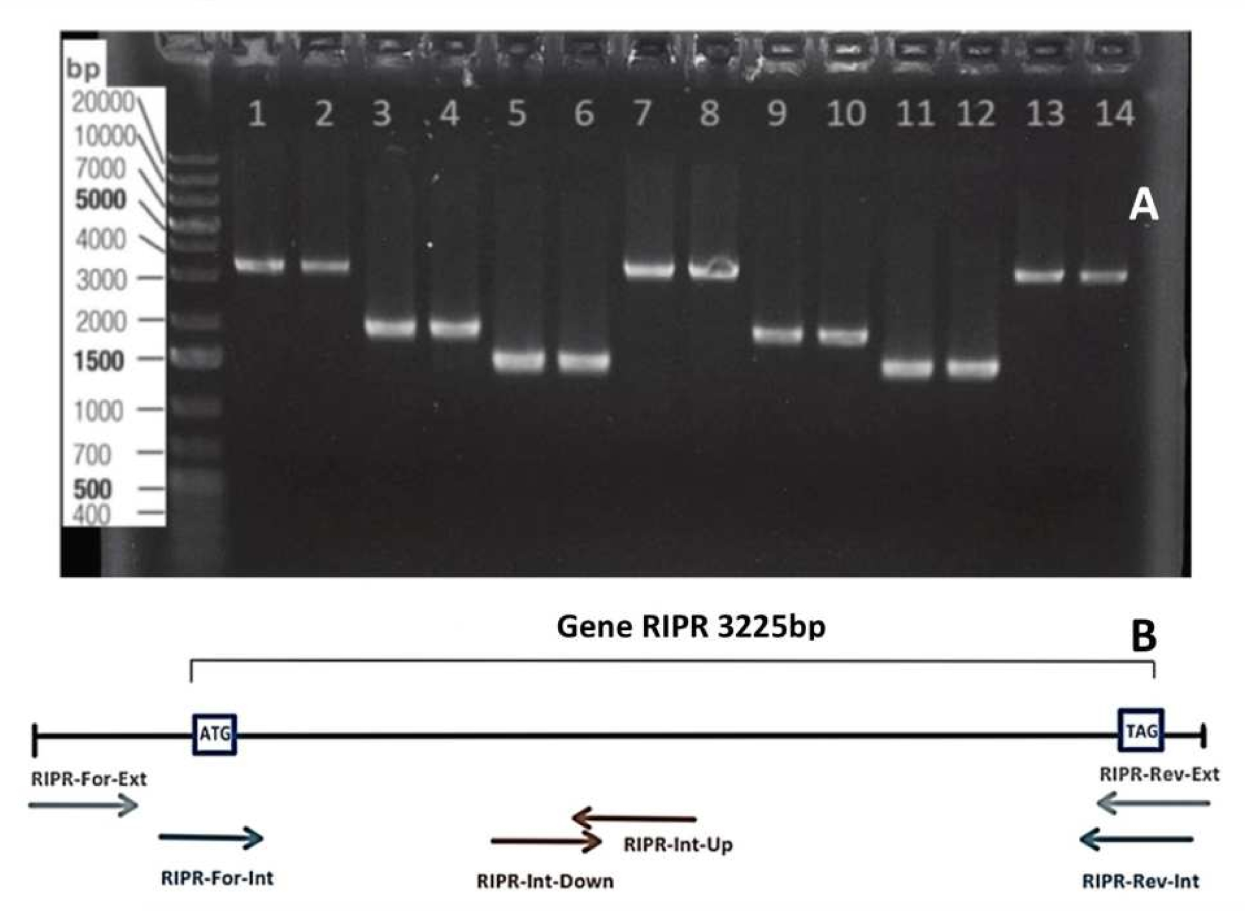


**Supplementary Figure 1.** A) 1% agarose gel electrophoresis showing the amplification performed on genomic DNA using specific primers for *pvripr* gene. Lanes 1, 2, 7, 8, 13 and 14 shows amplified products using primers RIPR-for-ext and RIPR-Rev-Ext. Lanes 3-4-9-10: by using primers RIPR-For-Int and RIPR-Int-Up a PCR product of 1990 bp was amplified. Lanes 5, 6, 11 and 12 shows amplified products if using primers. B) Layout of the primers described in Supplementary Table 1 on the *pvripr* gene.

**
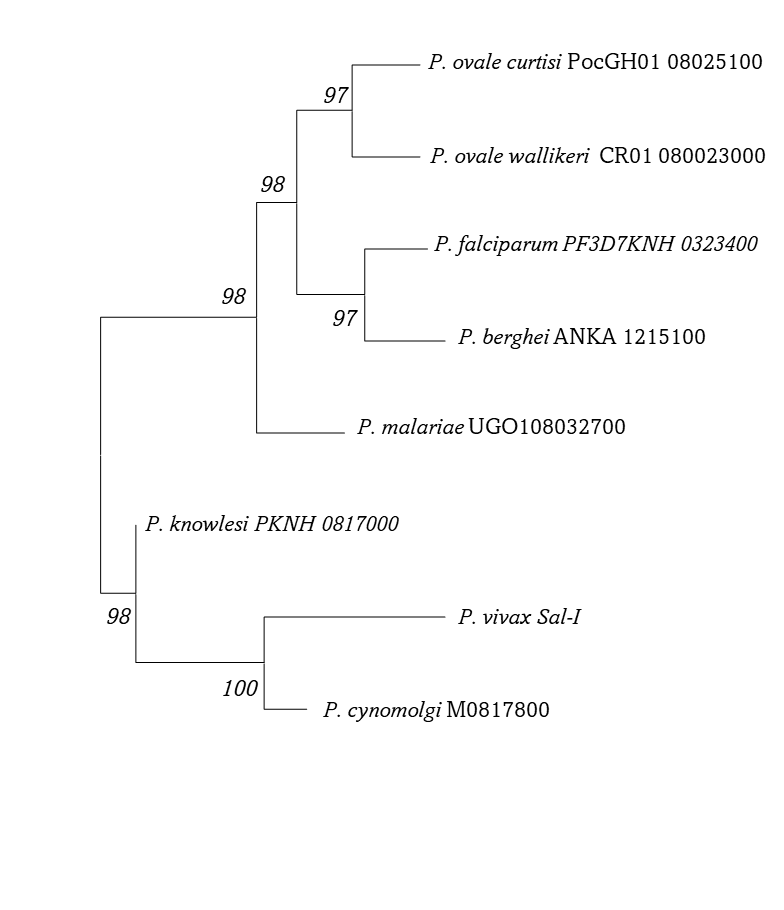
**

**Supplementary Figure 2.** Maximum likelihood phylogenetic tree from DNA sequences of RIPR homologues from different *Plasmodium* species based on the *ripr* gene sequence. This tree was constructed to confirm the orthologous relationship of PvRIPR with RIPR proteins from other *Plasmodium* species and to place PvRIPR within a broader interspecies evolutionary context. The tree shows two main branches, one of which comprises species most closely related to *P. vivax*, including *P. cynomolgi* (76.5% sequence identity) and *P. knowlesi* (76.8% sequence identity). The phylogenetic tree was built in MEGA v11 using Hasegawa–Kishino–Yano model with a proportion of invariant sites (HKY+I). Maximum likelihood trees were reconstructed with 1,000 bootstrap replicates.


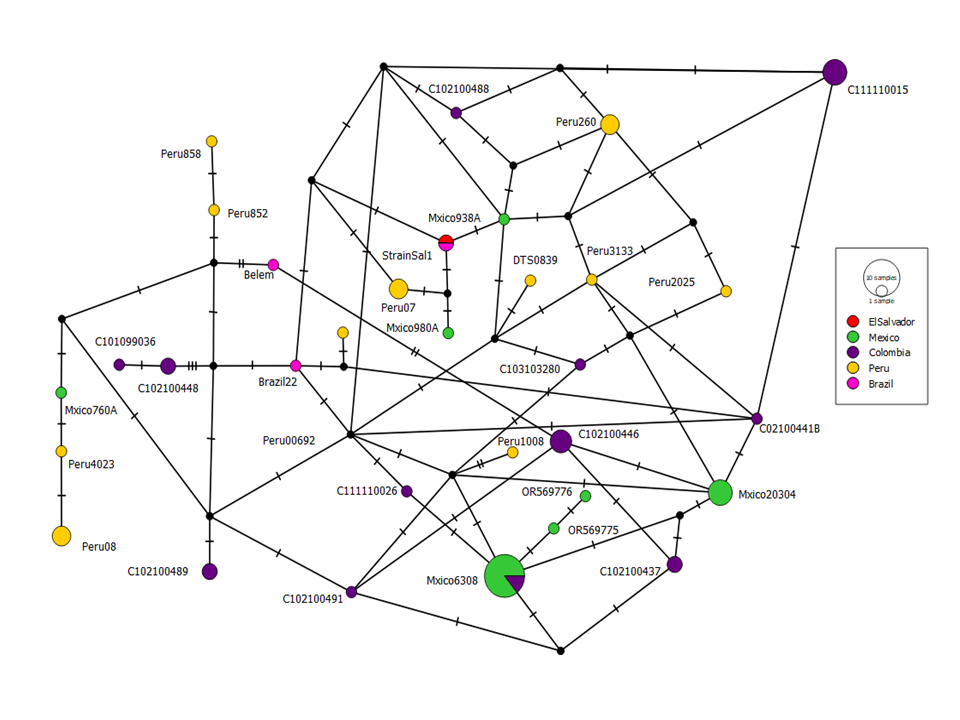


**Supplementary Figure 3**. Haplotype network of *P. vivax* isolates from Latin America using *ripr* gene sequence. The haplotype network shows that the haplotypes were separated by 1 to 3 mutational steps within them. The topology was reticulated and shows poor genetic structure. Only the highest frequent haplotype was shared between Mexico and Colombia. Each circle represents one haplotype, the size of the circle the number of sequences, and the vertical lines between haplotypes indicate the mutational steps. Black dots indicate no sampled or extinct haplotypes.


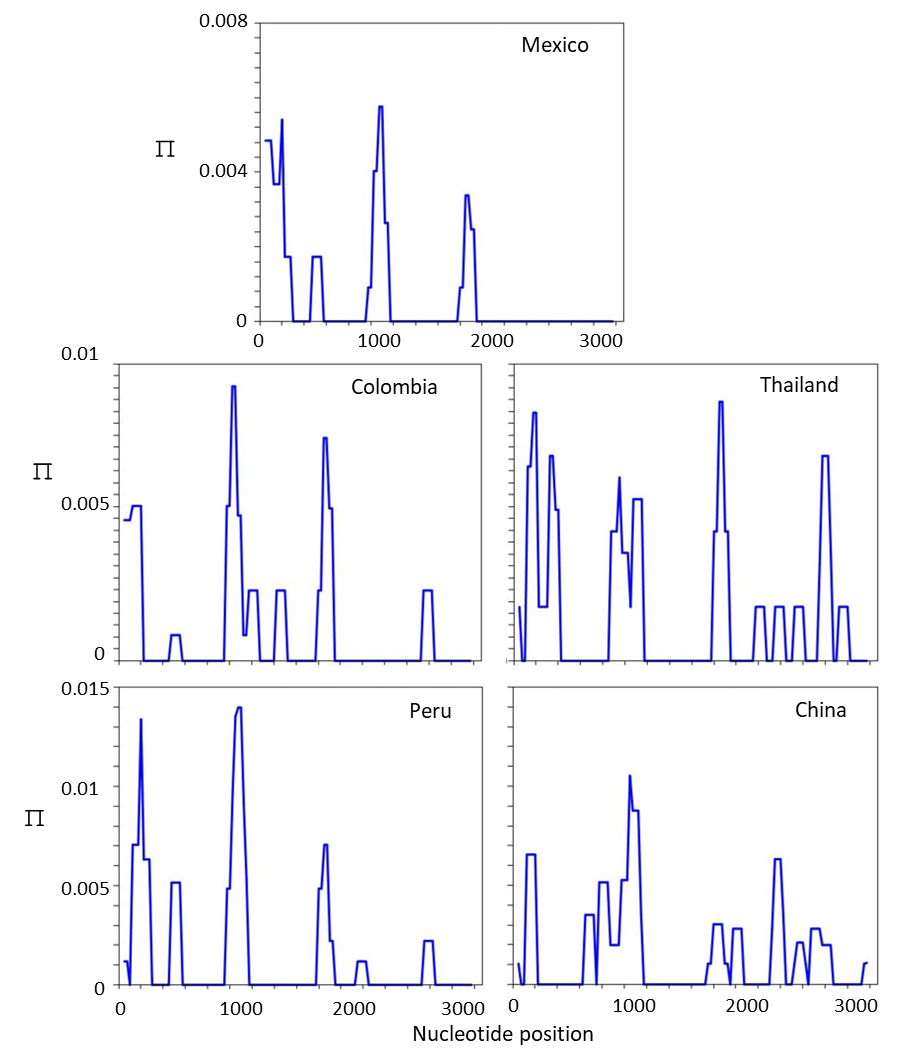


**Supplementary Figure 4**. Comparison of the slide window analysis of the nucleotide diversity (π) of *P. vivax* *rip*r gene from southern Mexico and other geographic locations. Window length of 100 bp and 25 overlapping nucleotides. In all locations diversity was present in the 5´gene segment, only in parasites from Thailand and China diversity on the 3´segment was more evident.

**
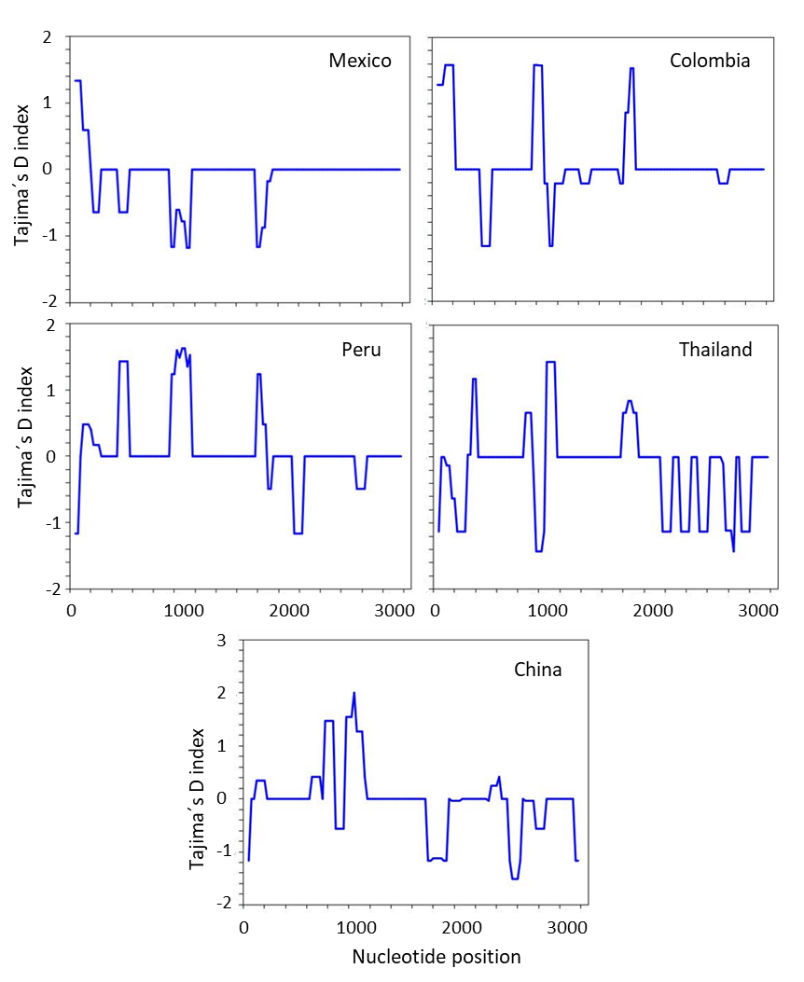
**

**Supplementary Figure 5.** Comparison of the slide window analysis of the Tajima´s D values of *P. vivax* ripr gene from southern Mexico and other geographic origins. Window length of 100 bp and 25 overlapping nucleotides (using as reference the Sal I strain). Consistently negative values were observed on the 3´of the gene segment, while positive values were mainly on the 5´gene segment.
